# Cistrome-GO: a web server for functional enrichment analysis of transcription factor ChIP-seq peaks

**DOI:** 10.1093/nar/gkz332

**Published:** 2019-05-04

**Authors:** Shaojuan Li, Changxin Wan, Rongbin Zheng, Jingyu Fan, Xin Dong, Clifford A Meyer, X Shirley Liu

**Affiliations:** 1 Shanghai Key Laboratory of Tuberculosis, Clinical Translational Research Center, Shanghai Pulmonary Hospital, School of Life Sciences and Technology, Tongji University, Shanghai 200092, China; 2 Department of Data Sciences, Dana-Farber Cancer Institute and Harvard T.H. Chan School of Public Health, Boston, MA 02215, USA; 3 Center for Functional Cancer Epigenetics, Dana-Farber Cancer Institute, Boston, MA 02215, USA

## Abstract

Characterizing the ontologies of genes directly regulated by a transcription factor (TF), can help to elucidate the TF’s biological role. Previously, we developed a widely used method, BETA, to integrate TF ChIP-seq peaks with differential gene expression (DGE) data to infer direct target genes. Here, we provide Cistrome-GO, a website implementation of this method with enhanced features to conduct ontology analyses of gene regulation by TFs in human and mouse. Cistrome-GO has two working modes: solo mode for ChIP-seq peak analysis; and ensemble mode, which integrates ChIP-seq peaks with DGE data. Cistrome-GO is freely available at http://go.cistrome.org/.

## INTRODUCTION

Insight into the biological roles of transcription factors (TFs) can be acquired through analyses of the ontologies of the genes that they regulate. Chromatin immunoprecipitation followed by high-throughput sequencing (ChIP-seq), is a broadly used technique for identifying TF binding sites genome wide (1). Despite the popularity of ChIP-seq technology and the importance of functional enrichment analysis few web servers are available for functional enrichment analysis based on ChIP-seq peaks (Table 1). Both ChIP-Enrich (2) and Enrichr (3) use a binary value to measure each gene’s potential to be regulated by a TF. Enrichr designates a gene as a TF target when the gene's transcription start site (TSS) is the nearest TSS to at least one of the TF’s ChIP-seq peaks. ChIP-Enrich assigns a gene as a TF target when there is at least one ChIP-seq peak located within 10 kb of the TSS, and using logistic regression and the Wald test for functional enrichment analysis. GREAT (4) differs from ChIP-Enrich and Enrichr, analyzing GO term associated genomic intervals. Although these web servers have been widely used, they have several limitations. First, for ChIP-seq data with many peaks (e.g. 20 000), tools based on binary value assignments could fail to identify the relevant enriched GO terms, due to too many genes being assigned as targets. Second, their functional enrichment analysis approaches rely on given sharp yet arbitrary cutoffs for target assignment (e.g. within 10 kb to a TSS). Third, none of the published web servers facilitate the integration of differential gene expression (DGE) data, such that derived from perturbation in the TF’s activity. This integration can improve the accuracy of the target gene prediction and thereby improve the GO association analysis.

**Table 1.** Summary of web servers for GO enrichment analysis of TF ChIP-seq data

Web server	ChIP-seq peaks and genes association method	Measurement of genes as targets	Integrating expression	GO enrichment calculation	Ref
Cistrome-GO	RP score depending on the distance to TSS	continuous values	Yes	Minimum hypergeometric test	
GREAT	Within a gene’s regulatory domain	0/1 assignments to genomic regions	No	Binomial test over genomic regions	(4)
ChIP-Enrich	Option from: nearest TSS, nearest gene, ≤1 kb from TSS, ≤5 kb from TSS, etc.	0/1 assignments	No	Wald test for logistic regression	(2)
Enrichr	Nearest TSS	0/1 assignments	No	Fisher’s exact test	(3)

To avoid the limitation of binary value assignments, we have previously developed a model that integrates the number of ChIP-seq peaks and their distances from a gene’s TSS in a real valued estimate of the TF’s potential for regulating that gene (5). In addition to ChIP-seq technology, information about a TF’s potential for regulating a gene can be derived from differential expression data upon the perturbation of a TF, such as stimulation, inhibition, knock-down or knock-out. Previously, we developed a software package BETA, which combined the regulatory potentials (RPs) defined by ChIP-seq peaks with relevant DGE data to improve the inference of the TF’s direct target genes (6). Here we present Cistrome-GO, a website version of BETA with enhanced features that conduct functional enrichment analyses of gene regulation by TFs in human and mouse. Cistrome-GO ranks genes by their likelihood of being the direct TF targets instead of selecting targets using an arbitrary cutoff and performs GO and pathway enrichment analysis based on gene ranks using the minimum hypergeometric (mHG) test (7,8). With a table of gene RP ranks and tables of GO and pathway enrichment as output, the Cistrome-GO web server gives biologists a deeper understanding of TF function.

## MATERIALS AND METHODS

### Calculation of adjusted regulatory potential score

**Figure 1.**
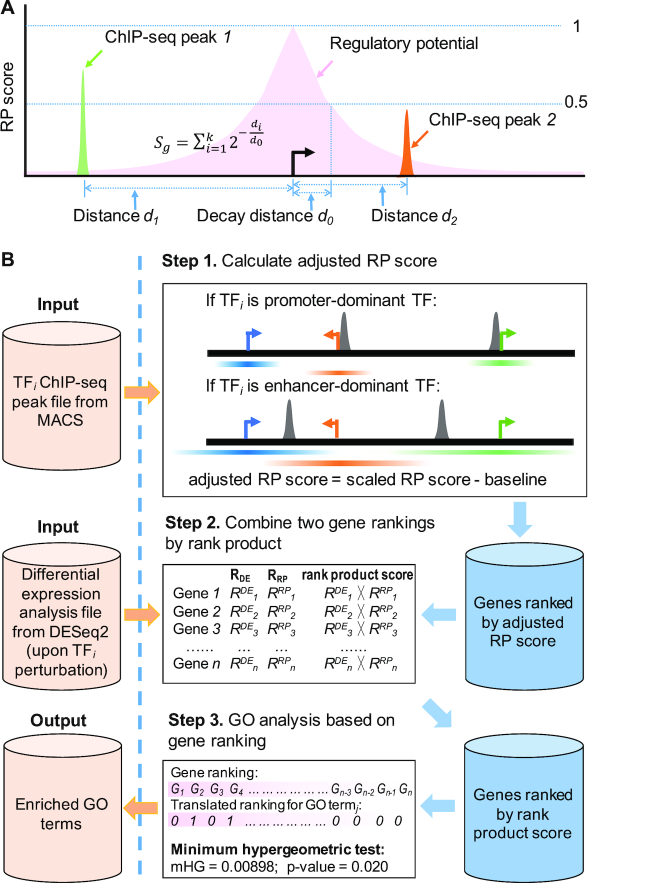
The workflow of Cistrome-GO. (**A**) Schema of RP score calculation for gene *g*. Two peaks are located near to gene *g*, with peak to TSS genomic distances of *d_1_* and *d_2_*. All *k* ChIP-seq peaks near the TSS of gene *g* are used in the RP score calculation (*k* = 2 in this figure). The pink area represents the decay function used in the RP score calculation. The parameter *d_0_* is the decay distance of the peak weighting function. (**B**) The ensemble mode workflow. If the user uploads both TF ChIP-seq peak and DGE analysis files, Cistrome-GO will perform an ensemble mode analysis based on the integration of the two types of data with the following three steps. Step 1: calculation of the adjusted RP score. Step 2: integration with differential expression data by rank product. Step 3: GO and pathway analysis based on gene ranking. Given a GO or KEGG term *j*, the gene ranking (with high ranking genes represented in bright pink) is translated into a series of *1*s or *0*s, which indicate the presence or absence of the ranked genes in the *j*th term. The mHG test is applied to this series to assess whether the *1*s tend occur near the top of the ranked gene list.

The RP score of a gene reflects the likelihood of the TF being a direct regulator of that gene. In Cistrome-GO, the RP score of each gene is defined as the weighted sum of peak contributions, where the weights decay exponentially with distance to the TSS (Figure 1A). The parameter *d_0_* is the decay distance of the weight function, with a default value of 1 kb for promoter-dominant TFs and 10 kb for the enhancer-dominant TFs. All *k* peaks near the TSS of gene *g* (within the distance of 15*d_0_*) are used in the calculation, and *d_i_* is the distance between the *i*th peak's center and the TSS. In the advanced settings users can override the default decay rate parameter *d_0_*.

Some genes tend to display higher RP scores than others across many TF cistromes. To quantify this bias, we calculated RP scores for each gene for 8470 and 6243 TF ChIP-seq datasets in human and mouse, respectively. For each ChIP-seq dataset, the RP scores for all genes were scaled to the range in [0, 1] by min–max normalization. We define the baseline RP score of a gene as the median value of its scaled RP scores in all processed ChIP-seq data in human and mouse, respectively. The genes with higher baseline RP scores are enriched in certain biological processes, such as regulation of intracellular signal transduction (Supplementary Figure S1). To mitigate this bias, for an uploaded ChIP-seq peak file, an adjusted RP score (i.e. the scaled RP score minus the baseline RP score) is calculated for each gene. The gene rank *R^RP^* is derived by decreasing adjusted RP score.

### Cistrome-GO working modes and implementation

Cistrome-GO is freely available at http://go.cistrome.org/. Cistrome-GO was implemented using the Django framework and an HTML/JavaScript interface. The source code is available at https://bitbucket.org/liulab/cistrome-go/src/master/. Cistrome-GO has two working modes. If the user provides both a TF’s ChIP-seq peak file in Browser Extensible Data (BED) format (such as MACS2 (9) output) and a DGE file (such as DESeq2 (10) output), Cistrome-GO will perform an ensemble mode analysis based on the integration of the two types of data (Figure 1B). If the user uploads only a TF ChIP-seq peak file, Cistrome-GO will perform the analysis in solo mode (Supplementary Figure S2).

### Classification of TF as promoter-dominant or enhancer-dominant type

A TF can regulate the expression of its target genes by binding cis-regulatory elements, including promoters and enhancers. For some TFs, such as E2F1, most binding sites are close to the TSS of target genes (<1 kb). Such TFs are of the promoter-dominant type. On the other hand, the binding sites for TFs such as AR and ESR1 tend to be less enriched in promoters, and those TFs are of the enhancer-dominant type. When a user uploads a TF’s ChIP-seq peak file, Cistrome-GO calculates the percentages of the most significant ChIP-seq peaks (top 2000, 5000, 10 000 and all peaks separately, if applicable; ranked by −log_10_(*P*-value)) within 1 kb of the TSS. If any of the calculated percentages is larger than a threshold, the TF will be classified as promoter-dominant. Otherwise, it will be classified as enhancer-dominant. We calculated the percentage of peaks close to TSS for 8470 human TF ChIP-seq data. Based on the distribution of the percentages, we set 20% (which is close to the median value 21.0%) as the threshold between promoter- and enhancer-dominant TFs. In Cistrome-GO, promoter-dominant and enhancer-dominant TFs have different parameters to calculate RP scores by default. Users can override these default settings in the advanced options.

### Integration with differential expression data by rank product

If the user provides a DGE analysis file in addition to a ChIP-seq peak file, an additional gene rank *R^DE^* is derived based on the *P*-value representing the significance of differential expression upon the TF’s perturbation. In the ensemble mode, the two ranks (*R^RP^* and *R^DE^*) are integrated by rank product. For example, if the ChIP-seq and DGE ranks of gene *i* are *R*_*i*_^*RP*^ and *R*_*i*_^*DE*^, respectively, its rank product score is *R_i_*^RP^ R_i_*^DE^*. The genes at the top of the list tend to have higher adjusted RP scores, together with more significant gene expression changes upon the TF’s perturbation; therefore, they are more likely to be true direct targets of the TF. In the settings users can opt to analyze upregulated genes, downregulated genes, or all genes. In the ensemble mode, Cistrome-GO also reports a receiver operating characteristic curve and precision-recall curve to assist the users to evaluate the association between ChIP-seq peaks and differential expressed genes. These curves are calculated on the basis of differentially expressed gene defined true positives and true negatives, with predictions based on adjusted RP scores.

### GO and pathway analysis based on gene ranking

In the ensemble mode, the gene rank is based on the rank product score, while in the solo mode, the gene rank is based on the adjusted RP score. In both modes, the top ranked genes are most likely to be directly regulated by the TF. In order to determine the GO terms (11,12) and KEGG pathways (13) that are significantly enriched in the genes at the top of the list in a threshold-free way, Cistrome-GO applies the mHG test on the rank for each GO or pathway term (7,8). To avoid reporting less informative GO terms, which are too general to provide useful information for TF’s regulatory function, those linked to more than 2000 genes have been excluded from the analysis. GO terms associated with <10 genes, too few for a reliable analysis, are also excluded. If two GO terms are highly similar, with a Jaccard index >0.85, only the GO term with the larger gene set is kept. An enrichment score, *P*-value and FDR is calculated for each GO or pathway term, and only the terms and pathways with FDRs <0.2 are reported.

### Performance evaluation of functional enrichment analysis

To compare functional enrichment results between Cistrome-GO and published web servers in a quantitative way, we compiled a list of standard Biological Process (BP) GO terms for the evaluated TFs as follows. Among the BP terms annotating the evaluated TF in AmiGO, we removed the processes with more than 2000 genes and <10 genes, and the remaining ones are regarded as the standard BP terms for the TF. As the set of GO terms used in Cistrome-GO is not identical to those used in other web servers, to make a fair comparison of their performance, the comparisons presented in this manuscript are limited to BP terms within the GO set used in Cistrome-GO. Given a functional enrichment analysis from Cistrome-GO or any of the other websites, the 10 most significant BP terms in a GO set used in Cistrome-GO are selected. For each of these top enriched terms, the semantic similarity between it and each of the standard BP terms is calculated using GOGO (14), and the maximum semantic similarity (MSS) is used to measure the similarity between the enriched BP term and the TF BP standard terms. The average MSS for the top 10 BP terms then represents the performance of the functional enrichment analysis: the higher the average MSS, the better the performance. Code and data for performance evaluation are at https://doi.org/10.6084/m9.figshare.8006285.

## RESULTS

### Cistrome-GO solo mode

To demonstrate the use of Cistrome-GO, we first display its output in solo mode (i.e. without integration of DGE data). MYOD1 is known to function as a master regulator in muscle cell differentiation, and most of its annotated BP GO terms are related to muscle development. Given a MYOD1 ChIP-seq peak file from myoblast (15), Cistrome-GO reported the enriched GO and pathway terms for gene regulation by MYOD1. When using all 43 057 ChIP-seq peaks, the 10 topmost enriched BP terms include ‘cytoskeleton organization’ and ‘skeletal muscle cell differentiation’ which are closely related to MYOD1’s regulatory function (Supplementary Figure S3A). Furthermore, if users wish to identify the putative target genes associated with a particular GO term, they can click the links in the ‘Genes’ column to see the adjusted RP scores and ranks of these genes (Supplementary Figure S3B). With the reported results, Cistrome-GO can assist users to reveal the regulatory functions of MYOD1.

**Figure 2.**
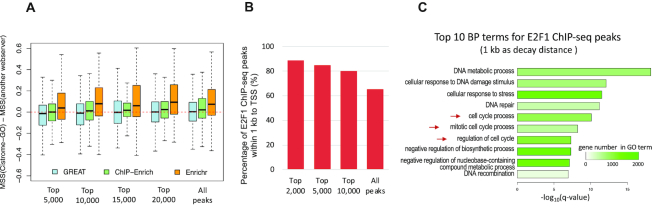
The performance of Cistrome-GO in solo mode. (**A**) Evaluation of the performance between Cistrome-GO and other web servers (including GREAT, ChIP-Enrich and Enrichr) on 256 TF ChIP-seq datasets. For each web server, the most significant 5000, 10 000, 15 000, 20 000 and all ChIP-seq peaks were used in the evaluation separately. The MSS score quantifies the similarity between the web server predictions and the standard set of GO terms for each TF. (**B**) The percentage of the most significant E2F1 ChIP-seq peaks (top 2000, 5000, 10 000 and all peaks separately; ranked by −log_10_(*P*-value)) are close (<1 kb) to TSS. (**C**) The top 10 enriched BP terms for 10 000 E2F1 ChIP-seq peaks using 1 kb as the decay distance. The BP terms indicated by red arrows are those relevant to cell-cycle functions. The color gradient represents the gene number in each GO term.

To quantitatively compare the performance of Cistrome-GO, GREAT, ChIP-Enrich and Enrichr, we considered all human TFs with ChIP-seq datasets available in the Cistrome Data Browser (cistrome.org/db). For each TF, if there is more than one available ChIP-seq dataset, the one with the largest number of peaks was used in the comparison. We compiled a list of standard BP terms for each TF (see ‘Materials and Methods’ section for details), and the TFs with over 10 standard BP terms were kept. In total, 256 TF ChIP-seq datasets were used in the comparison (Supplementary Tables S1 and 2; TF ChIP-seq data were deposited at https://doi.org/10.6084/m9.figshare.8006198). For each TF ChIP-seq dataset, we used the top 5000, 10 000, 15 000, 20 000 and all peaks (ranked by −log_10_(p-value)) of the ChIP-seq peak file as input for each web server separately, and we calculated MSS scores between web server reported top 10 enriched BP terms and the standard BP terms (see ‘Materials and Methods’ section for details). Based on the comprehensive comparison, the median performance of Cistrome-GO is better than that of ChIP-Enrich and Enrichr, and it is comparable to that of GREAT (Figure 2A). Besides, Cistrome-GO usually reports the results within 1.5 min, which is similar to GREAT and Enrichr, but much faster than ChIP-Enrich (usually over 10 min). Taken together, the solo mode Cistrome-GO performs well for functional enrichment analysis based on ChIP-seq peak files using an approach that is complementary to other methods.

### Promoter-dominant type TF

A TF can regulate the expression of its target genes by binding cis-regulatory elements, including promoters and enhancers. Some TFs, such as E2F1, are of the promoter-dominant type and tend to bind close (<1 kb) to the TSSs of genes (see ‘Materials and Methods’ section for details). For promoter-dominant TFs, the decay distance used to calculate adjusted RP scores is defined to be smaller than for the enhancer-dominant ones to recapitulate their promoter-related regulatory role. In Cistrome-GO, the default decay distance parameter is 1 kb for promoter-dominant TFs and 10 kb for enhancer-type TFs. In this study, an E2F1 ChIP-seq peak file from a prostate cancer cell-line LNCaP (16) was used to evaluate whether using 1 kb, instead of 10 kb, can improve the performance of Cistrome-GO for promoter-dominant TFs. For the given dataset, over 80% of top 10 000 ChIP-seq peaks are located within 1 kb of a gene's TSS (Figure 2B), confirming E2F1 is a typical promoter-dominant TF. E2F1 is known to regulate cell cycle genes. When using 1 kb as the decay distance, among the top 10 most enriched BP terms, 3 of them are relevant to cell cycle functions, including ‘cell cycle process’, ‘mitotic cell-cycle process’ and ‘regulation of cell cycle’ (Figure 2C), while none of the top 10 most enriched BP terms are relevant to cell cycle functions when using 10 kb a decay distance (Supplementary Figure S4). Taken together, using a smaller decay distance for promoter-dominant TFs could benefit the user to reveal the relevantly local regulatory functions of the TF.

### Ensemble mode of Cistrome-GO

**Figure 3.**
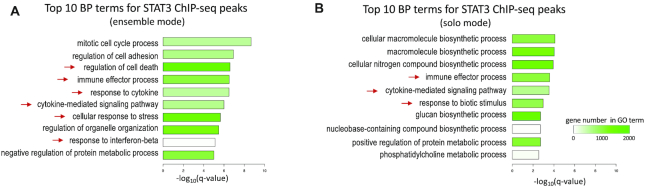
The performance of Cistrome-GO in ensemble mode, with STAT3 ChIP-seq and STAT3 knock-down DGE data as input. (**A**) The top 10 enriched BP terms in the ensemble mode. The BP terms indicated by red arrows are those relevant to STAT3’s reported functions. (**B**) The top 10 enriched BP terms for STAT3 in solo mode. The BP terms indicated by red arrows are those relevant to STAT3’s reported functions. The color gradient represents the gene number in each GO term.

Previously, we developed a software package BETA, which combined the RPs defined by ChIP-seq peaks with relevant DGE data to improve the inference of the TF’s direct target genes (6). In Cistrome-GO, if the user provides both a TF’s ChIP-seq peak file and a DGE analysis file related to a perturbation in the TF’s activity, Cistrome-GO performs an ensemble mode analysis based on the integration of the two data types. In this study, a STAT3 ChIP-seq peak file and a DGE file upon STAT3 knock-down in a breast cancer cell line (17) were used to evaluate whether the use of ensemble mode can improve the performance of Cistrome-GO. STAT3 is reported to regulate genes enriched in inflammation, immunity, and invasion in that study (17), and it is also reported to play a role in cell growth and apoptosis (18). Using the ensemble mode, of the 10 topmost enriched BP terms, 6 are relevant to STAT3 (Figure 3A), while in solo mode only 3 of the 10 most enriched BP terms are relevant to STAT3 (Figure 3B). Integration of DGE analysis with ChIP-seq peaks provides a better gene ranking of the putative TF target genes.

## DISCUSSION

Despite the popularity of ChIP-seq technology and the importance of functional enrichment analysis, few tools are available for functional enrichment analysis based on ChIP-seq peaks. Cistrome-GO has three unique features compared to published web servers. First, Cistrome-GO is the only tool to use continuous values instead of binary values to measure each gene's potential to be regulated by a TF based on ChIP-seq peaks. Tools based on binary value assignments of peaks to genes could fail to identify the relevant GO terms in cases with many ChIP-seq peaks, as they tend to assign too many genes as TF targets. Second, Cistrome-GO is the only tool with an option to integrate differential expression data upon a TF’s perturbation to improve the inference of the TF’s direct target genes. Third, Cistrome-GO performs GO term enrichment analysis based on gene rank instead of an unordered list of genes. In this way the genes are ranked by their likelihood of being the direct TF targets, instead of being selected using an arbitrary cutoff (e.g. within 10 kb of the TSS).

Cistrome-GO is a powerful and user-friendly website for functional enrichment annotation of TF regulation. With TF ChIP-seq data, and optional DGE data, Cistrome-GO outputs a table of gene RP ranks, and tables of GO and pathway enrichment. In summary, Cistrome-GO can give biologists a deeper understanding of TF function.

## Supplementary Material

gkz332_Supplemental_File
