# Supplementary material for: Cistrome-GO: a web server for functional enrichment analysis of transcription factor ChIP-seq peaks
Source: Nucleic Acids Res. 2019 May 4;47(W1):W206–11. doi: 10.1093/nar/gkz332 (PMC6602521; doi:10.1093/nar/gkz332)
Supplement: gkz332_Supplemental_File [file gkz332_supplemental_file.pdf]

Figure S1

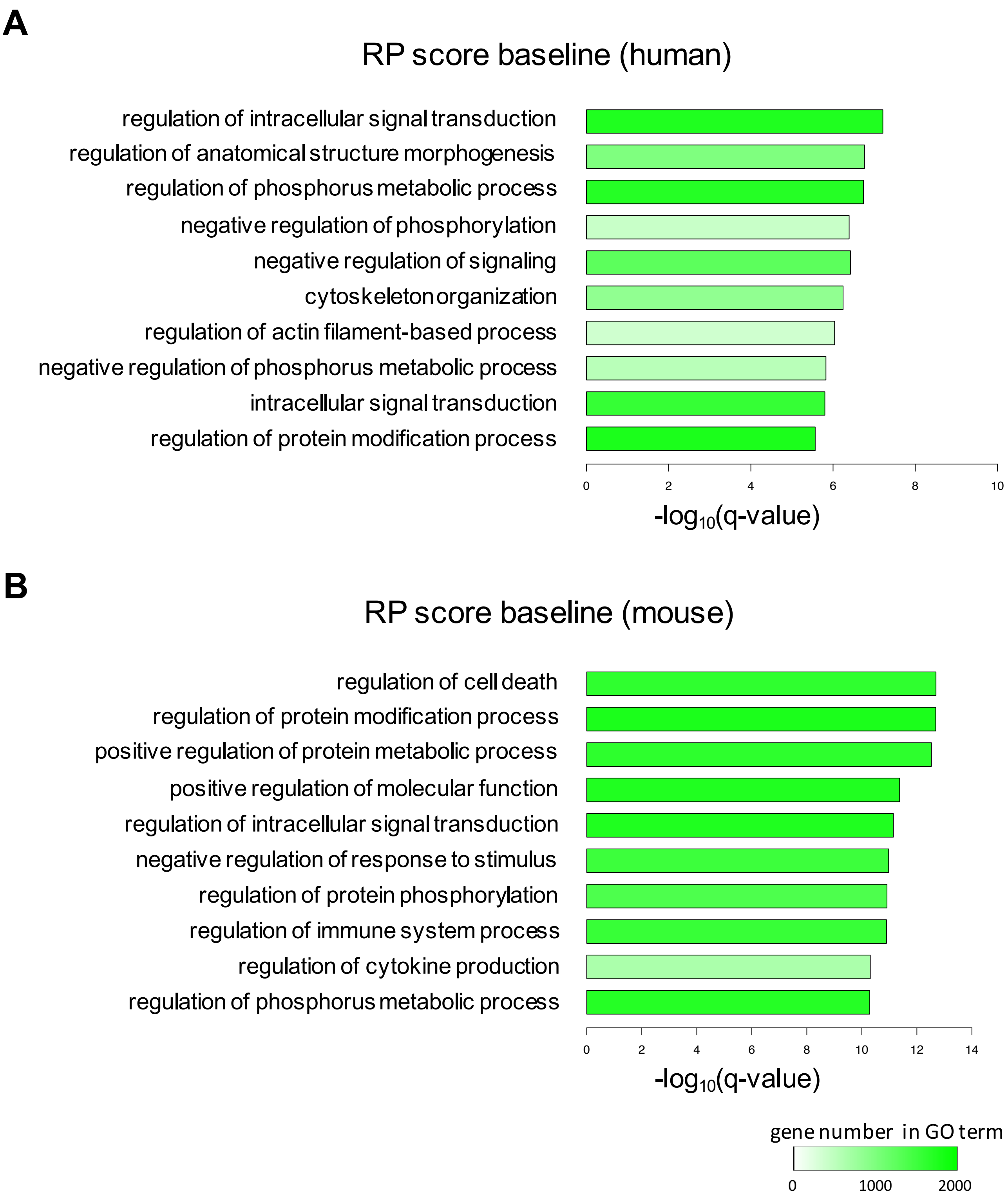

Figure S1. The top 10 enriched BP terms based on gene ranking by RP score baselines in human (A) and mouse (B) separately. The color gradient represents the gene number in each GO term.

Figure S2

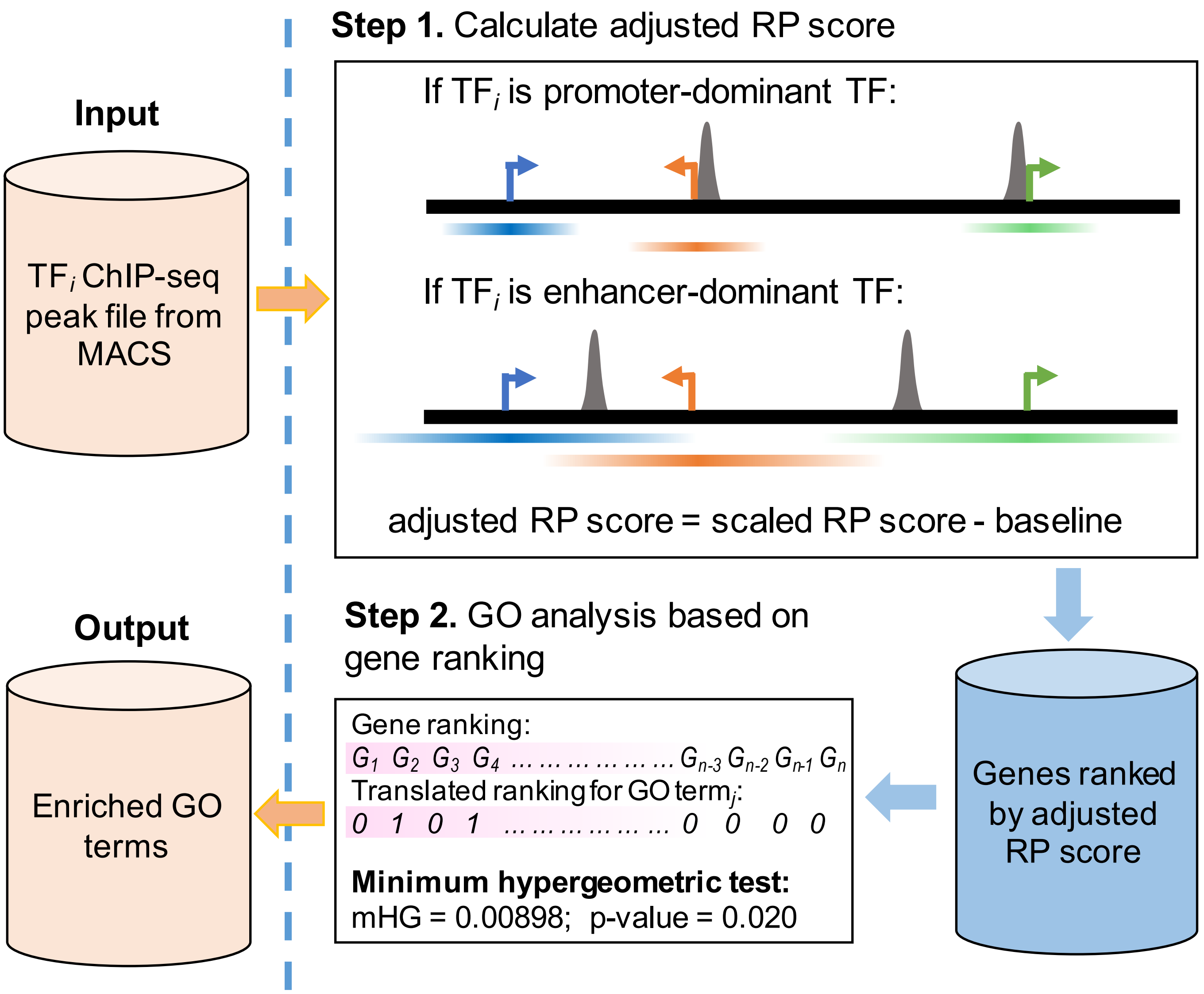

Figure S2. The workflow of Cistrome-GO (solo mode). If the user uploads only a TF ChIP-seq peak file, Cistrome-GO will perform a solo mode analysis with the following two steps. Step 1: calculation of adjusted RP score. Step 2: GO and pathway analysis based on gene ranking. Given a GO or KEGG term *j*, the gene ranking (with high ranking genes represented in bright pink) is translated into a series of *1*s or *0*s, which indicate the presence or absence of the ranked genes in the jth term. The minimum hypergeometric (mHG) test is applied to this series to assess whether the *1*s tend occur near the top of the ranked gene list.

Figure S3

A

GO terms	Enrichment(N,B,n,b)	P-value	FDR	Genes
anatomical structure formation involved in morphogenesis	1.866(16990,1614,705,125)	1.206E-10	5.799E-07	705
response to oxygen levels	2.661(16990,1221,301,55)	3.058E-09	7.353E-06	301
cytoskeleton organization	2.154(16990,665,854,72)	2.038E-08	3.268E-05	854
response to endogenous stimulus	1.762(16990,1397,1013,138)	5.214E-08	6.270E-05	1013
skeletal muscle cell differentiation	3.831(16990,1913,51,22)	9.606E-08	9.241E-05	51
response to abiotic stimulus	1.651(16990,1918,1038,178)	1.450E-07	1.163E-04	1038
hair cycle	19.219(16990,422,17,7)	6.680E-07	4.590E-04	17
regulation of locomotion	2.038(16990,672,944,73)	8.396E-07	5.048E-04	944
cell differentiation	1.594(16990,1971,1831,286)	9.890E-07	5.286E-04	1831
regulation of anatomical structure morphogenesis	1.816(16990,1797,999,160)	1.127E-06	5.419E-04	999

B

Gene	Coordinate	Visualize	Peak number	RP score	adjusted RP score	Ranked by adjust RP score
BHLHE40	chr3:4979411-4985180	WashU Browser	12	4.947	0.812	8
ID1	chr20:31605282-31606514	WashU Browser	18	4.974	0.777	19
EPHA2	chr1:16124336-16156109	WashU Browser	19	4.606	0.738	36
ARHGAP22	chr10:48446024-48605131	WashU Browser	13	4.164	0.696	69
CAPN2	chr1:223701592-223776018	WashU Browser	14	4.792	0.692	75
CDK5R1	chr17:32487086-32491253	WashU Browser	15	4.03	0.676	99
ACVR1	chr2:157736445-157838278	WashU Browser	13	3.78	0.634	158
CHRD	chr3:184380072-184389835	WashU Browser	13	3.624	0.607	224
PLXNB2	chr22:50274978-50307572	WashU Browser	11	3.596	0.602	242
PRICKLE1	chr12:42458337-42589770	WashU Browser	9	3.541	0.594	259

Figure S3. Cistrome-GO analysis of MYOD1 ChIP-seq in solo mode. A) Ranked list of GO terms. B) List of genes associated with "anatomical structure formation involved in morphogenesis" along with their RP scores.

Figure S4

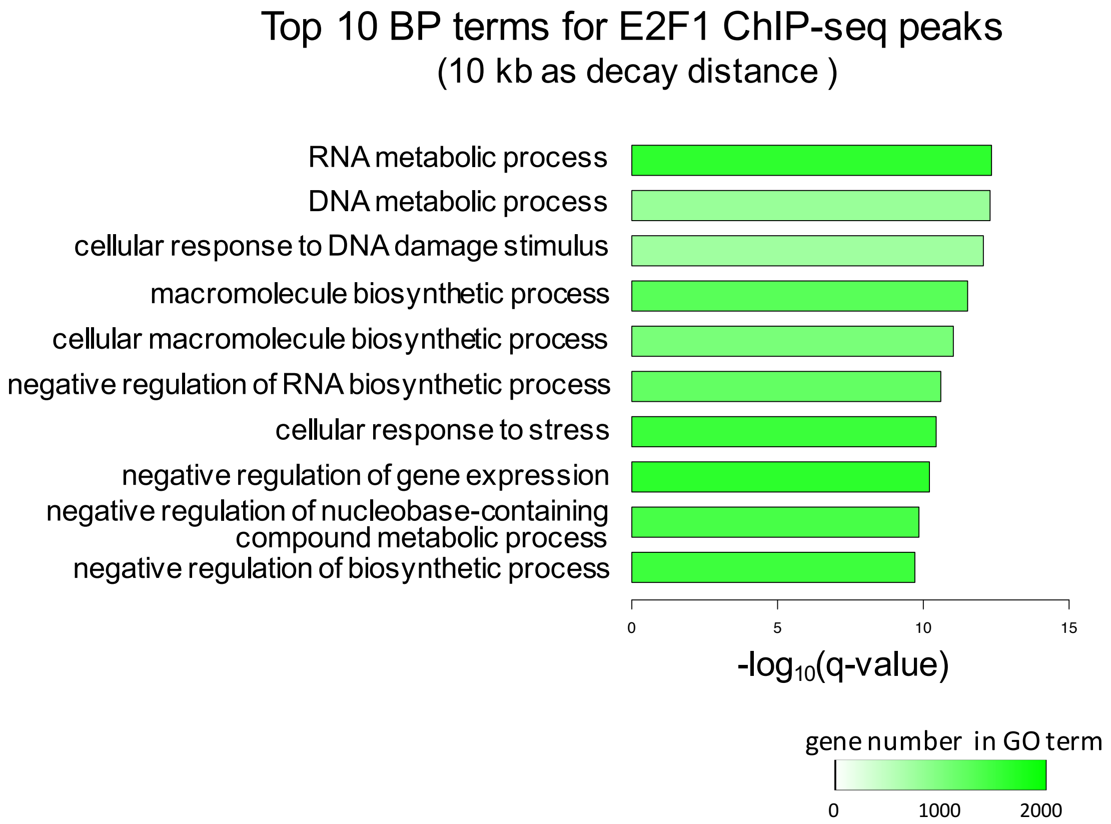

Figure S4. The performance of Cistrome-GO for the promoter-dominant TF, E2F1. The top 10 enriched BP terms for E2F1 by using 10 kb as decay distance. The color gradient represents the gene number in each GO term.
